# The Coding Logic of Interoception

**DOI:** 10.1146/annurev-physiol-042222-023455

**Published:** 2023-12-07

**Authors:** Ruiqi L. Wang, Rui B. Chang

**Affiliations:** Department of Neuroscience and Department of Cellular and Molecular Physiology, Yale University School of Medicine, New Haven, Connecticut, USA;

**Keywords:** interoception, interoceptive coding, sensory, vagus nerve, body–brain axis

## Abstract

Interoception, the ability to precisely and timely sense internal body signals, is critical for life. The interoceptive system monitors a large variety of mechanical, chemical, hormonal, and pathological cues using specialized organ cells, organ innervating neurons, and brain sensory neurons. It is important for maintaining body homeostasis, providing motivational drives, and regulating autonomic, cognitive, and behavioral functions. However, compared to external sensory systems, our knowledge about how diverse body signals are coded at a system level is quite limited. In this review, we focus on the unique features of interoceptive signals and the organization of the interoceptive system, with the goal of better understanding the coding logic of interoception.

## INTRODUCTION

Interoception, which broadly refers to the sensation of signals generated inside of the body and the subsequent regulation of physiological and behavioral functions, is a critical process for life and survival (1). It represents a bidirectional communication between internal organs and the central nervous system (CNS), in particular the brain. The primary goal of interoception is to provide the organism with a sense of its own internal state and the ability to perceive changes in this state. Different from most exteroceptive organs that are more specialized for specific external stimuli such as light, sound, and temperature, the interoceptive system surveys a diverse set of mechanical, chemical, and thermal signals from a large variety of internal targets including vasculature, bone, and body fluids. Such interoceptive information is crucial for maintaining body homeostasis, generating effective autonomic and voluntary responses to internal and external challenges, providing perceptual, emotional, and motivational drives, and modulating cognitive functions. Recent studies also revealed the close link between interoception and many neurological disorders including Alzheimer’s disease and Parkinson’s disease (2). Thus, the importance of the interoceptive system in overall well-being has been increasingly appreciated over the past decades.

In this review, we primarily focus on the sensory aspects of the mammalian interoceptive system and discuss sensations from visceral organs and vasculatures, especially those closely related to autonomic functions. Specifically, we summarize the historical views and recent breakthroughs of sensory coding, mainly in the respiratory, digestive, circulatory, and immune systems. We cover three main topics in the following sections: (*a*) the common and specific features of interoceptive signals across the body, (*b*) the organizational architecture of the interoceptive system that enables effective sensory coding and information processing, and (*c*) the unique properties of the interoception system (comparing to exteroception), our current understanding of the underlying biological solution, and future questions in this field.

## THE COMMON AND SPECIFIC PROPERTIES OF INTEROCEPTIVE SIGNALS

**Figure 1:**
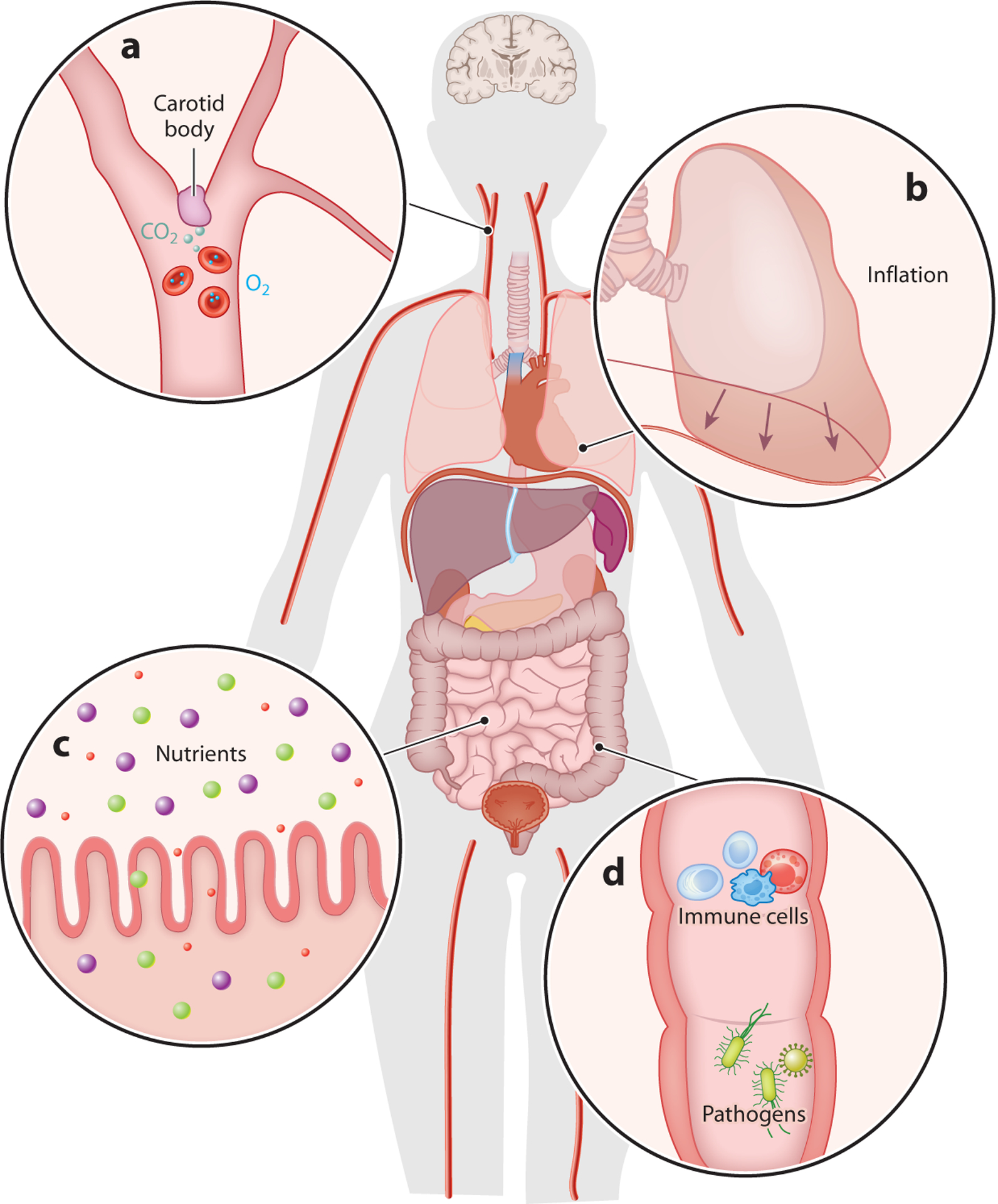
Interoception includes a large variety of body signals. Interoception is the sensation of signals originating within the body that represent physiological states. The interoceptive system responds to a wide range of mechanical, chemical, thermal, and pathological signals from internal organs and tissues, including (*a*) the detection of oxygen and carbon dioxide levels in the blood circulation, (*b*) the stretch or expansion of various organs (e.g., stretch of the lung during inflation is indicated by arrows), (*c*) nutrients from food, (*d*) and tissue damage, immune responses, and microbiota in the body. All of these internal signals and more can be sensed and monitored by the interoceptive system.

What needs to be sensed from the body? Internal states, such as circulation efficiency, nutrient level, energy expenditure, and immune balance, as well as organ changes under pathological conditions, are all generally considered as interoceptive signals (Figure 1). Some signals are primary, representing endogenous body status, such as circulating oxygen (O_2_), carbon dioxide (CO_2_), Na^+^, glucose, osmolarity, and specific pathogens. Others are secondary, released from specialized organ cells in response to pathophysiological changes, including various gut hormones, neurotransmitters, and cytokines. These individual signals are usually monitored by single dedicated sensors. However, to signal more complex internal changes such as food ingestion and sickness, many interoceptive signals with different sensory modalities often need to be integrated from different locations, sometimes even from multiple organs. For example, food ingestion will trigger a series of mechanical stretches along the gastrointestinal tract and change nutrient levels over time. These decomposed individual signals will be sensed in parallel from all related anatomical spots at different time points through multiple pathways, and they together inform the represented physiological change to the brain. In summary, a large collection of interoceptive signals with variable physical properties from diverse anatomical locations at different time points needs to be coded, processed, and integrated by the brain to reveal the underlying physiological meaning.

Each visceral organ has its unique anatomical structures, cellular compositions, and physiological roles. Therefore, it is somewhat expected that interoceptive signals from different organs may have organ-specific features and functions. For example, unlike most others, the respiratory system composed of the lung and airways is directly exposed to the air for gas exchange between the body and the environment. The airway filters, humidifies, and warms the air before it reaches the lungs. As a result, a number of air-related signals including pressure, temperature, gas composition, irritants, dust, airborne pathogens, and inflammatory mediators need to be constantly monitored (3). The digestive system comprising the gastrointestinal tract from mouth to anus and a few helping organs also faces a similar challenge: It is constantly exposed to external environments through ingestion. The digestive system performs two important functions, digestion and absorption, and is also the home to the gut microbiome. Signals related to ingestion, digestion, and absorption are closely monitored, including consumption volume, ingested contents such as water, salt, and nutrients, danger signals like toxins and pathogens, microbiome-derived metabolites, gut motility, and released digestive enzymes (4). Even within the digestive system, the esophagus and stomach are more exposed to undigested food, whereas the intestine mainly contacts digested molecules. Organs such as the pancreas only interact with filtered circulating factors.

Despite functional specificity, interoceptive signals from different organs might be physically identical. For example, mechanical stimulus is a common interoceptive signal in many organs. Interestingly, although at the macroscopic level mechanical stimuli may trigger distinct forms of tissue deformation in different organs, a family of mechanosensitive ion channels, PIEZO1 and PIEZO2, is widely employed to sense such mechanical changes all over the body (5). This seemingly surprising observation that one class of receptor could function under so many conditions is likely because only microscopic changes such as physical displacement of molecules and changes in cell membrane curvature can be directly sensed at the molecular level (6). Indeed, structural studies have clearly demonstrated that Piezo channels directly sense membrane curvature (7, 8). In addition to mechanical stimulus, there are other common interoceptive signals among multiple organs, such as inflammation and temperature. Moreover, some internally released messenger molecules, including hormones, biopeptides, and cytokines, can be used by different organs to convey distinct interoceptive information. For example, somatostatin is produced from many organs, mediating different functions (9). Within the pancreas, somatostatin is released from delta islet cells, inhibiting the release of pancreatic hormones. Somatostatin is also released from gastric D cells in the stomach and functions as an inhibitor of multiple gut peptides. In summary, unlike most exteroceptive signals, the correlation between the physical nature of an interoceptive signal and its physiological role can be complicated. While most current interoceptive studies have focused on individual organs, system-level cross-organ studies will provide valuable information about interoception.

## ORGANIZATION OF THE INTEROCEPTIVE SYSTEM

A primary goal of any sensory system is to convert environmental changes into electrical and biological signals, which can be further integrated and processed by the nervous system. Signal conversion is optimized during evolution to achieve better detection with improved sensitivity, selectivity, speed, and extraction of salient features. Such optimization serves to cope with the problems critical for sensory coding, including the physical nature of the stimulus, its intensity and frequency, and related spatial information. In addition, most sensory systems display some degree of plasticity to adapt to changing external and internal states. Various features of sensory signals then need to be appropriately decoded to generate meaningful physiological information. Moreover, multiple sensory modalities often need to be integrated to create a coherent perception. Altogether, these goals are achieved through a well-organized coding structure of each sensory system.

The interoceptive system can be generally divided into four major components: (*a*) primary sensory cells that initiate or sense environmental changes from the body, (*b*) sensory pathways that carry interoceptive information from the periphery into the brain, (*c*) a collection of brain centers for signal integration and processing, and (*d*) an effector arm that sends output signals from the brain to regulate physiology and behaviors. Many different cell types may serve as primary sensors for interoceptive signals, including (*a*) specialized organ cells such as enteroendocrine cells and organ intrinsic neurons (e.g., enteric neurons); (*b*) dedicated organ-innervating afferents with cell bodies in the jugular, petrosal, nodose, and dorsal root ganglia; and (*c*) CNS sensory neurons, such as the ones in circumventricular organs (CVOs) and the preoptic area (10) that sense circulatory factors and brain environment. There are two major routes that transmit interoceptive signals from the periphery into the brain, either through release of neuromodulators and hormones into the circulation or via organ innervating sensory neurons in the glossopharyngeal (cranial nerve IX), vagus (cranial nerve X), and spinal nerves. Many of these primary sensor cells can also receive signals from other primary sensor cells to present interoceptive signals in a coordinated manner to the brain. Through cross talk between cells at different levels, this multilevel organizational structure effectively increases coding complexity for interoception. Interestingly, most external signals including light, sound, smell, and taste are sensed through independently operated systems, while such highly dedicated modality-based structures are less apparent or largely absent in interoception. This structural difference between exteroception and interoception probably reflects the complexity of input signals. As most interoceptive signals are generated inside the body, their identity and dynamics are less variable and more predictable compared to exteroceptive signals from the external world and are coded via more hardwired mechanisms.

In the following sections, we review how interoceptive signals are sensed by primary sensor cells and discuss various body-to-brain pathways for interoception.

## SPECIALIZED ORGAN CELLS ARE PRIMED AS PRIMARY SENSOR CELLS IN INTEROCEPTION

**Figure 2:**
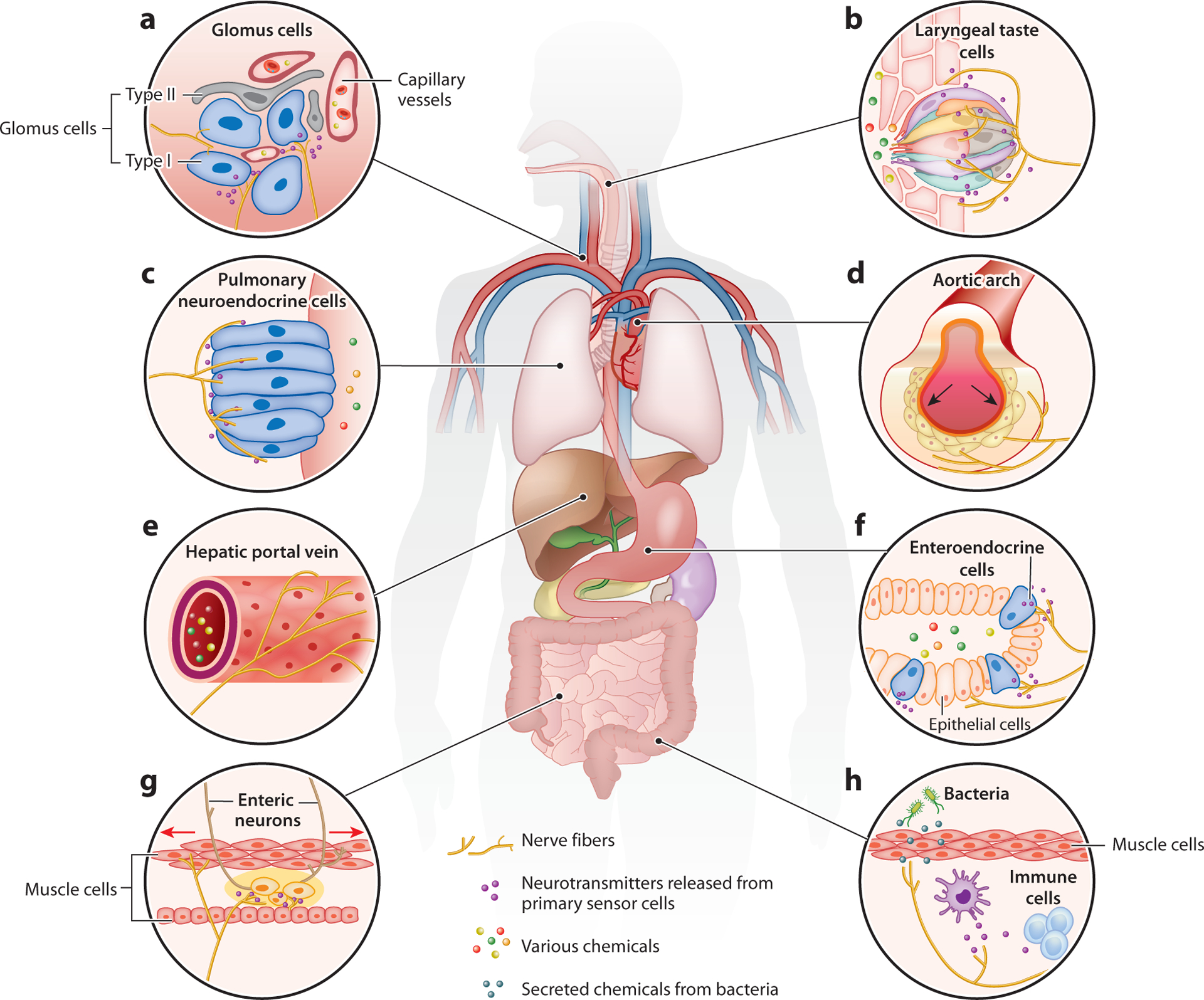
Primary interoceptive sensors. Primary interoceptive sensors can be specialized cells in visceral organs, sensory terminals of peripheral nerves, or sensory neurons in the brain. Depending on the particularity of different types of tissue, specialized organ-resident cells have well-adapted morphology and distribution and express distinct receptors that are specific to the type of stimulus they detect. When activated, these cells release neurotransmitters or neuropeptides to signal nearby peripheral sensory fibers via synaptic or paracrine mechanisms. Alternatively, they can release signaling molecules directly into the bloodstream, allowing for long-range communication throughout the body. Apart from these specialized sensory cells, sensory neurons in the nodose ganglia and dorsal root ganglia also send dense projections and form specialized endings in visceral organs. Examples of primary interoceptive sensor cells include but are not limited to the following. (*a*) Glomus cells in the carotid body are sensitive to the change in blood oxygen level, carbon dioxide level, and some circulatory hormones. Upon activation, they secrete ATP and a variety of neuropeptides that stimulate afferent sensory fibers. (*b*) Laryngeal taste cells detect particles and chemicals that enter the airway and secrete neurotransmitters including ATP to nearby vagal fibers. (*c*) Pulmonary neuroendocrine cells (PNECs) distributed at airway bifurcations respond to mechanical stimuli, hypoxic conditions, and allergens. PNECs also release abundant neurotransmitters and neuropeptides that excite lung-innervating nerves. (*d*) The aortic arch is densely innervated by the vagus nerve, which detects the change in aortic blood pressure. Arrows indicate that the mechanical force applies to the wall of the aortic arch when blood is pumped from the heart. (*e*) Sensory nerves on the hepatic portal vein are responsive to multiple hormones related to nutrient absorption. (*f*) Enteroendocrine cells (EECs) distributed in the epithelium of the gastrointestinal tract sense various contents from ingested food as well as tissue deformation. EECs contain different subtypes and release different signals in response to different stimuli, which modulate activities in the nearby nerves. (*g*) Sensory enteric neurons are local cells in the gastrointestinal tract that have the capacity to sense gastric volume change and nutrient composition. Enteric neurons can talk to the intraganglionic laminar endings from the vagus nerve and spinal nerve. Intramuscular arrays in the external muscular layers are mechanosensitive vagal endings that fire during gut distension. Arrows indicate stretch of the intestine wall. (*h*) Tissue barriers and specialized immune cells detect pathogens invading the body. Signal molecules involved in immune responses can also act on periphery sensory nerves. Peripheral sensory fibers can directly be activated by secretions of bacteria within the body.

Specialized organ cells are ideally positioned for sensing many body signals. They are broadly distributed in many visceral organs and function at the interface between different tissue types (Figure 2). These specialized organ cells often express corresponding receptors on their apical membrane to sense environmental changes. Upon activation, they release neurotransmitters or neuropeptides either to signal nearby neurons via synaptic or paracrine mechanisms or into the bloodstream for long-range communications via circulation. Here, we summarize the roles of a few important specialized organ cell types in interoception.

### Enteroendocrine Cells

Enteroendocrine cells are a group of specialized epithelial cells scattered throughout the epithelium of the gastrointestinal tract. With apical surface facing the lumen, enteroendocrine cells are primed to sense ingested food contents and tissue deformation (11–13). Upon stimulation, enteroendocrine cells release an array of gut hormones. A large collection of receptors within the G protein–coupled receptors (GPCRs) and transient receptor potential (TRP) channel families is expressed in enteroendocrine cells, including sweet taste receptors and sodium-coupled glucose transporters (SGLTs) for carbohydrates, FFAR1 (GPR40) and FFAR4 (GPR120) for fatty acids, calcium-sensing receptor (CASR) and metabotropic glutamate receptors for amino acids, olfactory receptor 558 (Olfr558) for microbial metabolites, and TRPA1 for irritants (13). Some enteroendocrine cells also express the mechanosensitive PIEZO2 channel to sense luminal forces (12, 14). As different nutrients trigger distinct hormone release patterns, it was traditionally thought that enteroendocrine cells are organized in labeled lines (one hormone, one cell type). Although enteroendocrine cells are historically categorized into different types based on the hormones they produce, including enterochromaffin cells (serotonin), I-cells [cholecystokinin (CCK)], K-cells [gastric inhibitory protein (GIP)], L-cells [glucagon-like peptide 1 and 2 (GLP1, GLP2); also peptide YY, PYY], X-cells (ghrelin), and others, recent single-cell studies demonstrated that enteroendocrine cells are more heterogeneous (15–19). Multiple sensory receptors can be expressed in one enteroendocrine cell type and many enteroendocrine cell types express multiple neurotransmitters and neuropeptides that can be differentially released under different conditions (18, 20–23). For example, enterochromaffin cells are polymodal chemosensors that monitor noxious molecules through the irritant receptor TRPA1, microbial metabolites through Olfr558, and mucosal forces through PIEZO2 (12, 22). These cells contribute to a myriad of gastrointestinal functions including contractility, transit pattern, visceral pain, and anxiety (12, 22, 24), yet it is unclear whether different functions are mediated by genetically distinct enterochromaffin cell subtypes or their different physical locations. An array of receptors for nutrients including carbohydrates, fats, and amino acids are broadly expressed in many enteroendocrine cell types, many of which have been implicated in nutrient sensing (11, 18). Interestingly, a recent study demonstrated that CCK^+^ neuropod cells use different receptors and neurotransmitters to signal luminal sugar (via glucose transporter SGLT1 and glutamate/CCK) and sweetener (via taste receptor T1R2/T1R3 and ATP). These new studies suggest that the coding logic for luminal signals in enteroendocrine cells is more complicated than previously thought and remains to be elucidated.

### Pulmonary Neuroendocrine Cells

Similarly, pulmonary neuroepithelial bodies, composed of clusters of pulmonary neuroendocrine cells (PNECs) located next to airway bifurcations, function as polymodal intrapulmonary sensors for inhaled O_2_ and CO_2_ levels, volatile odors, mechanical stimuli, and respiratory status (25). In vitro studies have demonstrated that O_2_ sensing in PNECs is through O_2_-sensing complexes including nicotinamide adenine dinucleotide phosphate (NADPH) oxidase and O_2_-sensitive K^+^ channels [possibly Kv3.3 and Kv4.3 (26)] on the plasma membrane (27–29). PNECs also express functional nicotinic acetylcholine receptors and can respond to nicotine exposure (30). Both in vivo and ex vivo experiments further demonstrated that PNECs can respond to mechanical stretch (31, 32), which under natural conditions could be generated by ventilation dynamics in the lung epithelium. PIEZO2 is expressed in PNECs and surrounding vagal sensory fibers (33), yet its role in PNECs has not been determined. PNECs express a number of signaling molecules including ATP, calcitonin gene-related peptide (CGRP), and serotonin to communicate with sensory nerves and surrounding cells including lung goblet and immune cells. Recent single-cell studies have shown that similar to enteroendocrine cells, PNECs are heterogeneous, expressing a diverse combination of sensory receptors and peptidergic genes (34), demonstrating the complexity of sensory coding in PNECs.

### Carotid Body Glomus Cells

The carotid body is a highly vascularized chemosensory organ at the bifurcation of the common carotid artery sensing information from the arterial blood and regulating cardiopulmonary functions (35–38). The carotid body contains two cell types: multimodal chemoreceptor cells (type I glomus cells) and supportive glial cells (type II glomus cells). Type I glomus cells are electrically excitable neuroendocrine cells surrounded by rich capillaries, sensing O_2_, CO_2_, pH, metabolites, and hormones from the blood through various receptors, ion channels, and signaling molecules. Glomus cells sense hypoxemia (low blood O_2_ level) through O_2_-dependent K^+^ channels and mitochondrial activity (37, 39), whereas hypercapnia (high CO_2_ level) sensing is achieved mainly through intracellular acidification catalyzed by carbonic anhydrase and subsequent inhibition of pH-sensitive K^+^ channels (40). Glomus cells can also be activated by hypoglycemia (low glucose concentration), possibly through inhibition of glucose-sensitive K^+^ channels and activation of an inward Na^+^ current (41, 42). Carotid glomus cells express several classes of voltage-gated, large-conductance, calcium-activated BK channels and background TASK-like K^+^ channels, but most are thought to be involved in sensory signaling (36, 43). In addition, glomus cells express leptin receptors and respond to leptin administration (44, 45). These stimuli ultimately trigger neurotransmitter release (46, 47) from glomus cells through a common calcium-dependent mechanism (48). Therefore, information on some signals can be integrated even at the level of glomus cells. For example, hypoxia and hypoglycemia have a synergistic effect on glomus cell cytosolic Ca^2+^ and neurotransmitter release (41, 49). As with enteroendocrine cells and PNECs, carotid glomus cells also express a large diversity of GPCRs (43), yet their heterogeneity and underlying coding logic remain to be determined. Moreover, although traditionally thought of as simply supporting cells, recent studies have demonstrated that type II glomus cells also contribute to chemotransduction and plasticity of type I cells (36).

Carotid body glomus cells synapse with sensory fibers and act as neurosecretory cells, producing a wide array of neurotransmitters and neuromodulators such as ATP, adenosine, dopamine, acetylcholine, and nitric oxide (NO). While ATP primarily acts as an excitatory neurotransmitter on postsynaptic ionotropic purinergic receptors (P2X2/3) in sensory nerve endings (50), other neuromodulators play a role in fine-tuning chemosensory afferent signals. For instance, dopamine inhibits depolarizing cationic currents in afferent fibers (51) and reduces Ca^2+^ current amplitude in glomus cells (52). In contrast, adenosine predominantly stimulates both pre- and postsynaptic cells (51, 53). Whether different neurotransmitters convey specific interoceptive information to the CNS is unknown. It is also unclear whether carotid body chemoreceptors code interoceptive signals differentially, as most if not all signals they respond to have the same physiological meaning. Conversely, reflexes mediated by carotid body chemoreceptors are highly dependent on actual physiological changes. For example, many conditions such as lack of atmospheric O_2_, hypoventilation, and apnea can lead to hypoxemia, and specific responses are generated under different conditions (38). It is possible that besides O_2_, carotid body chemoreceptors can sense and code factors differentially induced under these conditions. Alternatively, it is also hypothesized that condition specific responses are generated through central integration of information from both carotid body chemoreceptors and sensors at other locations.

### Immune Cells

The immune system constantly monitors pathogens and other outside invaders and protects the body using its own effectors. Immune cells can also signal the nervous system, providing immune-related interoceptive information to the brain. Organ-resident immune cells, such as mast cells, macrophages, and dendritic cells, detect the presence of pathogens by using different families of pattern-recognition receptors (PRRs) (54). Activation of PRRs initiates downstream signaling pathways by recognizing ligands. Each PRR detects pathogens from different origins and triggers specific signaling events that activate innate and adaptive immunity. For instance, RIG-I-like receptors (RLRs) are situated in the cytoplasm and detect viral genomic RNA (55), while Toll-like receptors (TLRs) are membrane receptors recognizing various molecules derived from microbes: TLR3/7/8/9 identify nucleic acids from viruses and bacteria (56); TLR4 recognizes lipopolysaccharide (LPS) (57); and TLR5, which is highly expressed by dendritic cells in the small intestine, identifies flagellin from flagellated bacteria (58). Activation of PRRs can initiate downstream signaling pathways, which can cause various effects such as the release and recruitment of cytokines, chemokines, hormones, and growth factors. For example, tumor necrosis factor alpha (TNF-α) and a variety of inflammation mediators are rapidly released upon TLR4 activation after LPS exposure (59). Additionally, TLR7 and TLR9 in dendritic cells lead to the production of interferons in response to viral infections, while TLR3 activation primarily triggers the secretion of cytokines such as interleukin (IL)-12p40 (60). Circulating immune cells also have a crucial role in detecting pathogens. For instance, monocytes present in the bloodstream express various TLRs and NOD-like receptors (61) and they can eliminate microbes, produce cytokines and chemokines, and present antigens to T cells (62). Cytokine receptors are broadly expressed in many sensory neurons, yet, so far, no synaptic structures have been described between immune cells and neurons, suggesting that their interactions are mainly via paracrine signaling. How different immune signals are coded in the nervous system is only beginning to be elucidated and our knowledge in this important field is rapidly growing (63–65).

### Sensory Neurons in the Organ-Intrinsic Nervous System

In addition to specialized organ cells, many visceral organs have their intrinsic nervous systems, such as the enteric nervous system on the gut (66), the intrinsic cardiac nervous system on the heart (67), and intrapancreatic neurons within the pancreas (68). These organ-intrinsic neurons are composed of sensory, motor, and interneurons, forming complex neural networks. Organ-intrinsic sensory neurons can directly sense organ signals. For example, the intrinsic primary afferent neurons (IPANs) of the enteric nervous system sense gut stretch, microbial metabolites, and inflammatory signals (66, 69). IPANs are categorized into a few different subtypes based on their gene expression profiles (70). Some IPANs express PRRs, including TLR2/3/4/7, and IPANs can respond to luminal bacteria within seconds, suggesting that they might be directly involved in bacteria sensing (71–73). Enteric neurons can also directly sense bacterial metabolites and endotoxins, such as excretory/secretory products from *Nippostrongylus brasiliensis*, and release neuromedin U to drive antiparasite immunity (74). Similarly, intrinsic cardiac neurons are reported to monitor cardiac cycles and contractility (75). Organ-intrinsic sensory neurons may also form synaptic connections with specialized organ cells, such as between enterochromaffin cells and enteric neurons (22), and indirectly sense organ signals. Similar to other organ-specific cells, organ-intrinsic neurons do not signal the brain directly.

## ORGAN-INNERVATING SENSORY NEURONS SENSE BOTH PRIMARY AND SECONDARY INTEROCEPTIVE SIGNALS

Sensory neurons within the glossopharyngeal, vagus, and spinal nerves constitute the second level within the body–brain axis. These neurons are pseudounipolar, with one axon innervating peripheral targets and the other projecting into the CNS. The cell bodies of spinal sensory neurons are in dorsal root ganglia (DRG) along the rostral-caudal axis of the spinal cord. Although generally considered as somatosensory neurons, the role of DRG neurons in visceral sensation has been recognized for a long time and increasingly appreciated over the past decade. As in somatosensation, visceral targets are innervated by anatomically proximal DRGs. For example, the heart is primarily innervated by C7-T4 DRGs (76), adipose fat is innervated by T11-L3 DRGs (77), and distal colon is innervated by L6-S2 DRGs (78). Centrally, DRG neurons project into the dorsal horn of the spinal cord at their corresponding levels. Interoceptive information is further transmitted into the brain through multiple spinal tracts. Sensory neurons within the glossopharyngeal and vagus nerves are in a series of cranial ganglia (jugular, petrosal, and nodose ganglia), which in mice are often fused together into the nodose-jugular-petrosal (NJP) ganglia (79, 80). These sensory cranial ganglia each have their preferred body targets. For example, jugular neurons primarily innervate the external auditory canal skin, cranial meninges, larynx, pharynx, and upper airways; petrosal neurons innervate the carotid body and carotid sinus; and nodose neurons innervate most visceral organs above the distal colon level, including cervical organs such as the trachea and esophagus, thoracic organs such as the heart and lung, and abdominal organs including the stomach, small intestine, pancreas, and transverse colon. Through the vagus and glossopharyngeal nerves, interoceptive signals are directly transmitted into the brainstem to the caudal region of the nucleus tractus solitarii (NTS). It is generally believed that interoceptive signals within the physiological range are carried through the vagus nerve while signals for more extreme pathological conditions are mediated by DRGs. However, there are many exceptions to this general rule, and the precise coding principle between the vagus nerve and DRG neurons in interoception remains to be addressed.

An important role of organ-innervating sensory neurons is to provide spatial information for interoceptive signals. Because the physiological meaning of an interoceptive signal depends not only on its physical nature but also heavily on its location in the body, such spatial information is critical for signal discrimination in the brain. Neurons in the jugular, petrosal, and nodose ganglia and DRGs at various spinal levels have spatially dedicated body targets, and such spatial information will be carried together with the coded signals and passed to the CNS (81–83). Simultaneous retrograde tracing from multiple body targets further demonstrated that sensory neurons in nodose ganglia and DRGs that project to different visceral organs are largely nonoverlapping (84–86), indicating that organ information is also coded within each ganglion. Interestingly, within ganglion, sensory neurons that innervate different visceral targets are not spatially clustered, and viscerotopic maps were not observed (85, 87–89). Instead, nodose neurons innervating different visceral targets are largely segregated based on their genetic features (85). These differentially expressed gene modules also constitute a visceral organ dimension coding organs along the body’s rostral–caudal axis. Intriguingly, a separate set of genes code tissue environment of sensory endings independent of visceral organs. Together these data suggest that spatial information of interoceptive signals is precisely coded in sensory ganglia by genetics.

Organ-innervating sensory neurons may function as primary sensors, sensing airway stretch, inhaled irritants, blood pressure fluctuations, intestinal inflammation, and many others (79). In other cases, they may receive interoceptive signals indirectly through specialized organ cells via paracrine or synaptic transmission. In the following sections, we discuss interoceptive coding by organ-innervating sensory neurons in several important physiological systems.

### The Lungs and Airways

The respiratory system is equipped with a variety of sensory receptors from the vagus nerve (3). Most of the mechanosensitive receptors in the airway are myelinated, and one myelinated axon may give rise to one or several unmyelinated arborized terminal structures that differ in complexity. Based on electrophysiological properties from single fiber recordings in response to lung inflation or deflation, vagal intrapulmonary mechanosensors can be classified as rapidly adapting receptors (RARs) and slowly adapting receptors (SARs). Functional studies have provided some clues for the anatomical locations of RAR and SAR endings; for example, RARs are likely within or beneath the epithelium in both intra- and extrapulmonary airways, and SARs are probably within the lung parenchyma around the smooth muscle (3). A variety of airway nerve endings have been described using immunocytochemistry against general neuronal markers; for example, arborized terminals around airway smooth muscle were observed near the local receptive fields of in vivo recorded SAR fibers (90). However, these traditional approaches make it extremely difficult to accurately link characterized electrophysiological properties with specific ending morphologies or molecular identities. As a result, the anatomical arrangement of RAR and SAR endings remains undetermined. It is not clear whether RARs and SARs are equipped with different sensory machineries or whether they are from the same group of neurons sensing mechanical changes from different locations. It is also speculated that the same vagal airway neurons may form both RARs and SARs and function as multisensors (91). Recent studies using mouse genetic tools showed that ablating *Piezo2* from nodose neurons significantly reduced vagal afferent responses to lung inflation, demonstrating that airway stretch is directly sensed by vagal sensory neurons through mechanosensitive ion channels (33). Functional studies using spatial transcriptomics coupled in vivo calcium imaging of nodose ganglia further demonstrated that distinct PIEZO2^+^ nodose neuron subtypes have different adaptation rates, which potentially represent SARs and RARs (85). Within the lung, PIEZO2^+^ lung stretch–sensitive nodose neurons terminate at bronchi bifurcations (85), structures with the highest shear stress during inspiration (92) that are thus ideal spots for sensing airway stretch.

Many vagal chemosensory afferent types contribute to asthma, cough, and allergic airway inflammation, most of which are capsaicin-sensitive TRPV1^+^ unmyelinated C-fibers. TRPV1^+^ vagal pulmonary afferents are traditionally thought to be primary sensors for inhaled irritants (3). Sulfolipid-1, a bacterial metabolite from *Mycobacterium tuberculosis*, also directly activates TRPV1^+^ NJP and DRG neurons and triggers cough in guinea pigs (93). In addition, TRPV1^+^ neurons also express a large variety of receptors for signals from surrounding sensor cells. MrgprC11, a previously identified itch receptor, is expressed in a subset of jugular neurons (94). MrgprC11 is critical for sensing allergic signals potentially released from mast cells and inducing bronchoconstriction and airway hyper-responsiveness after virus infection. A subset of lung-projecting vagal sensory neurons express Fc+R1 and directly respond to allergen immune complexes, such as ovalbumin-specific immunoglobulin E, to initiate type 2 airway inflammation (95). Sodium channel Na_V_1.8^+^ nodose neurons also express a number of cytokine receptors and respond to cytokines such as IL-5 in ex vivo cultures (96). A subgroup of TRPV1^+^ vagal neurons that express sphingosine-1-phosphate receptor 3 (S1PR3) are also responsible for asthmatic-like broncho-constrictions (97). A recent study demonstrated that petrosal TRPV1^−^/GABRA1^+^ neurons sense prostaglandin E2 (PGE2) released after influenza infection through PGE2 receptor 3 (EP3) and mediate systemic sickness responses (98), uncovering a novel role of TRPV1^−^ sensory neurons in pathogen sensing and sickness behaviors. Vagal P2RY1^+^ neurons, which are TRPV1^−^/GABRA1^−^, also mediate a series of airway defense mechanisms including apnea, vocal fold closure, swallowing, and expiratory reflexes (80, 99). P2RY1^+^/AGTR1A^+^ nodose neurons interact with clusters of specialized epithelial cells along the airways including lung neuroepithelial bodies and laryngeal taste buds (80, 85, 99). They sense ingested water and acids indirectly from laryngeal taste receptor cells via ATP signaling and trigger swallowing (80). Sensory fibers from the DRG neurons mainly innervate the large airways and are absent from the alveolar region (100). How DRG and vagal sensory neurons differentially contribute to interoception in the respiratory system remains to be elucidated.

### The Gastrointestinal Tract

Similar to the lung and airways, the gastrointestinal tract is monitored by the vagus and spinal sensory nerves with some differences in their distributions. While the entire gastrointestinal tract is innervated by DRG neurons at corresponding spinal levels, the vagus nerve densely innervates the esophagus, stomach, and intestine and is largely absent from the distal colon and rectum (101). Classical retrograde/anterograde tracing and electrophysiological recording studies have mapped three types of vagal sensory ending structures along the gastrointestinal tract, including intramuscular arrays (IMAs) running parallel to smooth muscle fibers, intraganglionic laminar endings (IGLEs) in close proximity to myenteric ganglia, and mucosal endings within the gut epithelium, with the highest density in the villi and crypts of the proximal small intestine (102). Electrophysiological recordings showed that vagal afferents respond to esophageal stretch independent of exocytosis with a very short latency (~6 ms) (103), suggesting that they are primary mechanosensors. Intriguingly, as determined using posthoc anterogradely staining, receptive fields of electrophysiologically identified vagal mechanosensitive afferents are close to IGLEs, indicating that IGLEs may serve as mechanosensors (103–105). Recent genetic studies involving retrograde labeling, single-cell RNA sequencing, and adeno-associated virus (AAV)-guided tracing in various Cre mouse lines have provided a genetic roadmap for vagal sensory neurons in the gastrointestinal tract (85, 87, 88). Consistent with electrophysiological studies, in vivo calcium imaging of vagal ganglia demonstrated that GLP1R^+^/PIEZO2^+^ nodose neurons that form IGLEs on the stomach sense gastric distension (85, 88). However, IGLEs are not the only gut mechanosensors. IMAs, which have the appropriate anatomical features for mechanosensation (106), respond to stomach stretch with rapidly adapting kinetics (85). Moreover, vagal mechanosensitive afferents in the gut may not necessarily be the primary sensor. Both PIEZO2^+^ enteroendocrine cells (12) and IPANs (66) can sense mechanical changes in intestine and signal gut afferents.

It is well documented that nodose neurons sense ingested food components from the intestine, including carbohydrates, amino acids, fats, and toxins. However, their sensory endings do not penetrate the intestinal epithelium and therefore have no direct contact with luminal contents. Instead, they receive signals from enteroendocrine cells through receptors for various neurotransmitters and neuropeptides including GLP1, CCK, PYY, glutamate, ATP, and serotonin. Multiple neurotransmitters and neuropeptides may be used for the same specialized organ cell-to-sensory neuron connection to transmit different interoceptive information. For example, recent studies revealed that intestinal neuropod cells use glutamate and CCK to signal sugar information at different timescales and use ATP for noncaloric sweetener (20, 21). Interestingly, intestinal neuropods also signal visceral pain through DRG neurons (107), although it is unclear whether they are the same cells that sense nutrients. It has been shown in somatosensation that gentle touch activates mechanosensitive DRG afferents through both direct (via the PIEZO2 channel) and indirect (through mechanosensitive PIEZO2^+^ Merkle cells) pathways (108, 109), demonstrating that second-level sensory neurons can serve as primary and indirect sensors simultaneously for the same signal. A similar mechanism also likely exists in interoception.

A variety of vagal sensory neuron subtypes have been revealed for sensing ingested nutrients and luminal components using in vivo calcium imaging of nodose ganglia. For example, GPR65^+^ neurons respond to a variety of nutrient, chemical, and osmolarity changes perfused through the duodenal lumen, with no clear preference among sugar, amino acid, and salt but some preference for fatty acids (88). Tachykinin 1–positive nodose neurons sense osmolarity changes from the hepatic portal area (110). Additional studies identified that 4–5% of nodose neurons respond specifically to sugar but not general osmolarity changes (111). A following study further showed that sugar-specific nodose neurons also respond to intestinal fat, suggesting that they might function as multisensors for nutrients (112). This study also uncovered a subset of nodose neurons (~8%) that only responds to intestinal fat but not sugar, suggesting that fat signals might be selectively coded within nodose neurons. Intriguingly, activating gut GPR65^+^ nodose neurons increases hepatic glucose production (113), suggesting a role of these neurons in glucose sensing as well.

In addition to IGLEs, IMAs, and mucosal endings, DRG neurons also form vascular endings associated with branch points of mesenteric arteries encoding both contraction and distension of the gut wall and traction on the mesenteries (114). These vascular afferent endings are also associated with other organs in the digestive system such as the pancreas. Single-cell RNA sequencing and calcium imaging of retrogradely labeled DRG neurons showed that, like nodose neurons, they are functionally heterogeneous (89). Interestingly, gut-innervating thoracolumbar and lumbosacral DRGs show distinct gene expression profiles, suggesting that a genetic program that codes spatial projection may also exist in the spinal sensory nerves (89). Extensive evidence has shown that DRG neurons are heavily involved in visceral pain (115). For example, DRG neurons directly sense a large variety of bacterial pathogens and metabolites through a collection of receptors including formyl peptide receptors, TLRs, and noxious receptors TRPV1 and TRPA1 (116). It has been clearly demonstrated that TRPV1^+^ DRG but not vagal neurons sense bacterial pathogen–derived signals from *Salmonella enterica* serovar Typhimurium (117). Interestingly, a recent study also revealed that PIEZO2 expressed in TRPV1-lineage DRG neurons senses colorectal distension and contributes to subsequently induced physiological and behavioral changes (118). Cross-organ sensitization has been extensively documented (84), in particular between the colon and bladder, which are sensed via DRG neurons at the same spinal levels, yet the underlying molecular and cellular mechanisms remain to be determined. In addition to visceral pain, DRG neurons can indirectly transmit nutrient information from ileum enteroendocrine cells via GLP1 signaling and contribute to appetite and food intake regulation (119), suggesting that DRG neurons also play an important role in hunger and satiety. The coding logic between the vagus and spinal sensory nerves, the two major physical body–brain connections, thus represents a major research topic in the field of interoception.

### The Cardiovascular System

Blood pressure fluctuation, represented by stretch of arterial wall at the carotid sinus and aortic arch, is directly sensed by the glossopharyngeal and vagus nerves respectively, through mechanosensitive PIEZO channels. Knocking out both *Piezo1* and *Piezo2* within PHOX2B^+^ NJP neurons almost completely abolished aortic depressor nerve response to blood pressure elevation and greatly dampened the baroreflex (120). Anatomically, PIEZO2^+^ nodose neurons form a macroscopic claw around the aortic arch, decorated with end-net endings embedded in collagen matrix (121). Nodose neurons also sense a variety of mechanical and chemical signals from the heart and regulate cardiopulmonary functions (122), yet the underlying mechanism remains unclear. The glossopharyngeal nerve receives additional signals from the carotid body glomus cells mainly through ATP and P2X2/P2X3 signaling via synaptic transmission (123). The precise identity of carotid body innervating NJP neurons still needs to be determined.

### Lymphoid Organs

Although interactions between sensory nerves and organ-resident immune cells have been extensively studied, much less is known about the interactions between sensory nerves and lymphoid organs. Early immunohistochemistry studies have suggested that both primary and secondary lymphoid organs including the thymus, spleen, and lymph nodes are innervated by nerve fibers expressing CGRP and substance P, which are sensory neuron markers used in many classical studies (124). A recent study using mouse genetic tools revealed dense innervation of popliteal lymph nodes by Na_V_1.8^+^ DRG neurons (125). Popliteal lymph node–innervating DRG neurons are genetically heterogeneous and also distinct from skin-innervating counterparts. Interestingly, within the lymph node, most sensory endings were observed close to the nodal capsule and only rare afferent fibers were seen in the deep cortex, suggesting that sensory neurons mainly interact with perivascular and subcapsular cells but not naïve lymphocytes. The precise immune signals sensed by DRG neurons remain unknown. It is also unclear whether lymph nodes at other locations or other lymphoid organs are similarly innervated.

## CENTRAL NERVOUS SYSTEM SENSORY NEURONS AND INTEROCEPTION

Body signals can also be directly sensed by sensory neurons in the brain through circulation. Some circulating factors represent endogenous body status, such as Na^+^, glucose, osmolarity, CO_2_, and pathogens, while others are released from specialized organ cells including various gut hormones, neurotransmitters, and cytokines. Although the vast majority of the brain is isolated and protected by the blood–brain barrier (BBB), some structures around the ventricles (e.g., CVOs) lack the complete BBB and thus are capable of communicating with the rest of the body through blood circulation (126). CVOs are divided into two groups based on their functions. The secretory CVOs include the subcommissural organ, pituitary neural lobe, median eminence, and pineal gland. The sensory CVOs include the organum vasculosum of the lamina terminalis (OVLT), subfornical organ (SFO), and area postrema (AP). Because of fenestrated capillaries and loosely apposed glial cells in the sensory CVOs, circulating factors including blood-borne hormones, cytokines, and pathogens can penetrate the parenchyma and be sensed by CVO sensory neurons. Both the SFO and OVLT are located within the cerebrum: SFO on the midline rostral wall and OVLT along the anterior wall of the third ventricle. The AP is a brainstem nucleus at the caudal end of the fourth ventricle. On the one hand, all three sensory CVOs are thought to sense some common signals, including cytokines and pathogens. This is somewhat expected because these circulating factors are presented to brain sensory neurons in a similar manner. On the other hand, clear functional segregation among sensory CVOs has been observed as well. Both the OVLT and SFO are critical for sensing blood osmolarity, sodium level, and angiotensin, as well as for regulating thirst, sodium appetite, and blood pressure (127, 128). The AP has been extensively associated with nausea, vomiting, and sickness behaviors, as well as food intake, cardiovascular regulation, and energy homeostasis (129–134). One explanation for such functional specificity is that sensory CVOs express unique subsets of receptors. For example, an atypical sodium channel Na_X_ that potentially mediates sodium sensation is selectively expressed in the SFO and OVLT but not the AP (135, 136). In contrast, GFRAL, the receptor for growth differentiation factor 15, is selectively expressed in the AP (132). Another possibility is that the unique anatomical structures of sensory CVOs including capillary organization, cell components of endothelial barriers, and distribution patterns of tight junction proteins may also contribute to their sensory specificity. Interestingly, BBB permeability may vary within the sensory CVO (137). For instance, the vasculature structure is not homogeneous in the SFO, with less-permeable nonfenestrated vasculature mainly in its shell and highly permeable fenestrated capillaries restricted at its core (138). Whether these anatomical differences lead to functional zones in the SFO is unclear. Moreover, unlike most other brain sensory nuclei, the AP also receives direct projections from vagal afferents, suggesting that it might be primed for signal integration from multiple pathways.

Some other brain nuclei can also function as interoceptive sensors. For example, the arcuate nucleus of the hypothalamus next to the third ventricle is anatomically primed to sense a variety of nutrients and adiposity hormones and serves as an integrator of energy states (139, 140). The highly permeable environment of the median eminence enables effective perfusion of circulatory factors to its nearby arcuate nucleus (140). Moreover, under certain circumstances such as during fasting, a specialized ependymal cell type that maintains the blood–hypothalamus barrier named tanycyte can also undergo reorganization and actively transport circulatory molecules to the arcuate nucleus (141). In vivo multiphoton imaging demonstrated that ghrelin, a well-known hunger hormone mainly released from stomach in response to hunger, is able to penetrate the fenestrated capillaries in the arcuate nucleus (142). Functional studies have also shown that circulating ghrelin directly activates arcuate agouti-related protein (AGRP)/neuropeptide Y neurons to promote food intake (143–145). On the other hand, arcuate AGRP neurons are potently inhibited by circulating leptin, a satiety hormone released from adipose cells (146–148). Although the underlying mechanism is still under debate, recent studies using a CRISPR-based knockout strategy suggested that this inhibitory effect is direct through leptin receptors expressed on AGRP neurons (147).

Some brain sensory cells can directly sense BBB-permeable factors from circulation. For example, it has been recognized for more than a century that central respiratory chemoreceptors exist to sense accumulated CO_2_ in the brain and regulate breathing via the central respiratory reflex (149–151). Although their exact locations remain elusive, recent studies have pointed to the retrotrapezoid nucleus (RTN) in the brainstem as a prototypical central respiratory chemoreceptor. RTN neurons are activated by CO_2_ both in vitro and in vivo (152), driving respiratory efforts by regulating breathing frequency. Optogenetic activation of RTN neurons potently stimulates breathing (153), while ablating RTN neurons abolishes brain acidification induced respiration changes (154). It is generally thought that RTN neurons sense CO_2_ mainly via pH changes. Some studies also suggest that CO_2_ molecules and bicarbonates might be sensed directly as well (155, 156). Several molecular and cellular mechanisms by which RTN neurons sense brainstem CO_2_ have been proposed and investigated, including (*a*) via TASK-2 and GPR4, which are cell-autonomous pH sensors, and (*b*) via paracrine signals such as ATP from nearby astrocytes that function as primary sensors for pH and CO_2_. Interestingly, specialized microvasculature within the RTN may help local CO_2_ accumulation by reducing blood flow, thus facilitating CO_2_ sensing in the RTN (157), demonstrating a link between anatomical structures and physiological functions.

In addition to circulating factors that inform body status, the cerebrospinal fluid (CSF) contains molecules representing environmental changes within the CNS (158). The role of the CSF in immunological protection of the CNS has been increasingly appreciated over the past decade. A variety of related biomarkers and cytokines within the CSF have been used for diagnosis of brain disorders including Alzheimer’s disease, multiple sclerosis, and CNS infection (64, 159, 160). Some cells are primed to sense information from the CSF. For example, *Pkd2l1*^+^ CSF-contacting neurons lying along the central canal of the spinal cord extend their ciliated terminals into the ventricular cavity through ependymal cells (161). These neurons monitor pH and pressure changes within the spinal CSF and regulate posture and locomotion (161–163). A recent study in zebrafish also showed that CSF-contacting neurons respond to brain bacterial infection and promote host survival (164), demonstrating the importance of CSF sensing in maintaining CNS health.

## CENTRAL REPRESENTATION OF INTEROCEPTION

**Figure 3:**
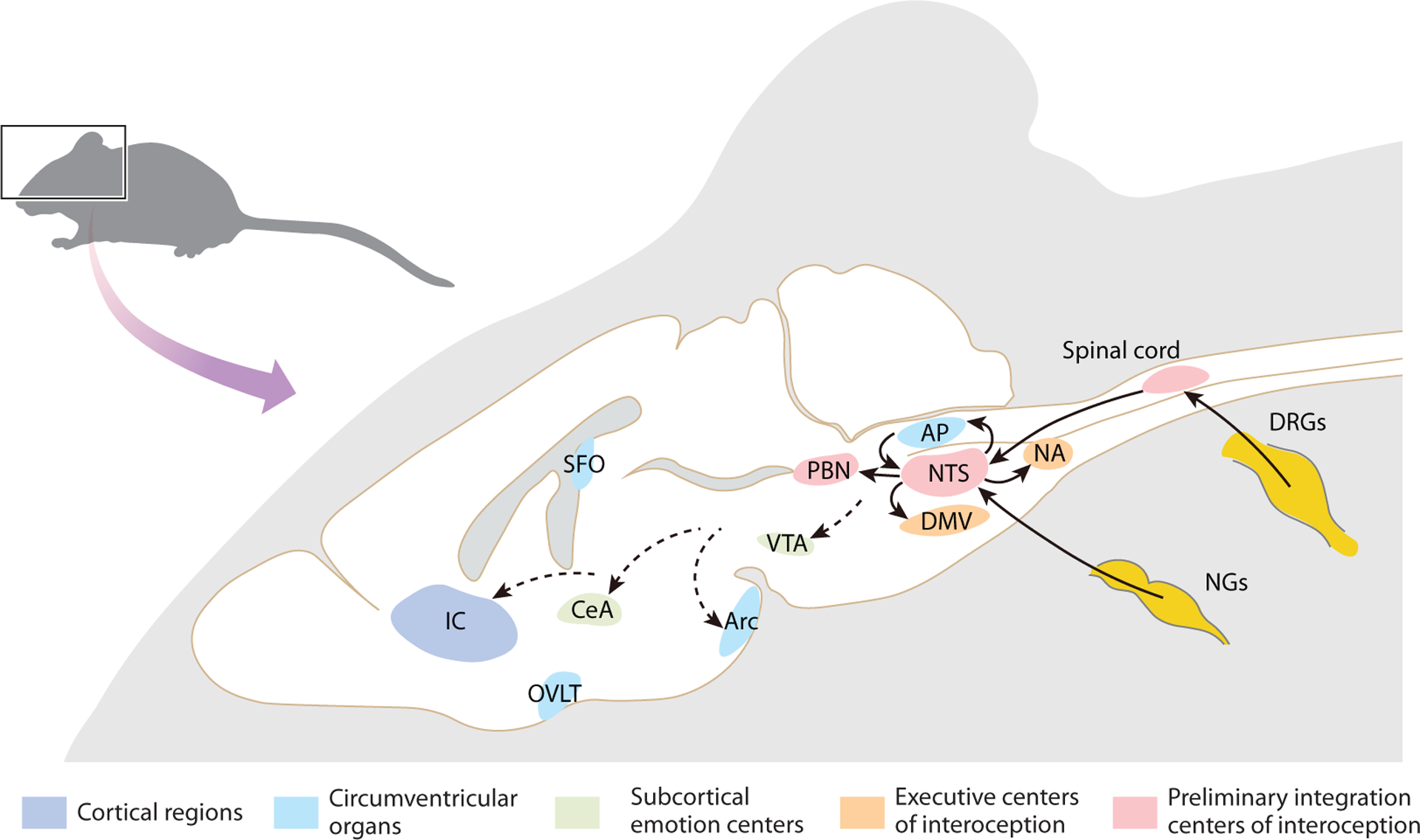
Central representation of interoception. The NTS integrates information related to internal organs and coordinates autonomic responses by activating motor neurons in the DMV and NA. Along with the vagus nerve, sensory neurons in DRGs also send interoceptive information through the spinal cord to the NTS or directly to higher-order centers such as the PBN. In addition to rapid autonomic responses, some interoceptive signals that are processed in the NTS can also be transmitted to higher-order centers, such as the PBN, PVN, LRN, and PAG. After further processing, the PBN sends integrated information to various brain regions, including reward centers such as the VTA, emotion centers such as the CeA, and the insular cortex, which is believed to generate perceptive responses. The solid arrows indicate direct projection, whereas the dashed arrows represent indirect connection. CVOs with distinct BBB structures such as the AP, SFO, OVLT, and some other paraventricular nuclei also function as interoceptive sensors that extract certain information from the circulation, such as the Arc. These special sensors in the brain receive and integrate information from the periphery; e.g., a subpopulation of vagal sensory neurons also projects to the AP directly. Furthermore, signals with biological significance generated in these regions can also be further distributed to autonomic centers such as the NTS and integrated into the higher-order regions mentioned above. Abbreviations: AP, area postrema; Arc, arcuate nucleus; BBB, blood–brain barrier; CeA, central amygdala; CVO, circumventricular organ; DMV, dorsal motor nucleus of the vagus; DRG, dorsal root ganglion; IC, insular cortex; LRN, lateral reticular nucleus; NA, nucleus ambiguus; NG, nodose ganglion; NTS, nucleus of the solitary tract; OVLT, organum vasculosum of the lamina terminalis; PAG, periaqueductal gray; PBN, parabrachial nucleus; PVN, paraventricular nucleus; SFO, subfornical organ; VTA, ventral tegmental area.

Interoceptive information is processed and widely distributed in many brain regions (Figure 3). The caudal NTS represents one of the first brain centers for interoception, receiving direct visceral information from the vagus and glossopharyngeal nerves (82). Therefore, the caudal NTS likely plays a similar organizational role as the dorsal lateral geniculate nucleus in vision and the rostral NTS in taste. Traditionally it was thought that such structures are simply relays for sensory information in the brain, with little or no signal integration and processing, but extensive recent studies have shown that such understanding is not correct. Apart from direct inputs from vagal sensory neurons, the NTS also receives interoceptive information from the spinal cord lamina I (165), as well as humoral information from the adjacent AP (166). In addition, the NTS is capable of directly sensing some blood-borne hormones such as leptin (167). Genetically distinct vagal subpopulations have different projection patterns in the NTS (33, 87, 88, 99, 168). For instance, vagal GLP1R^+|^ neurons that function as gastric mechanoreceptors extensively project to the medial NTS, whereas GPR65^+^ neurons that sense duodenal luminal contents preferentially target the commissural NTS (88). Vagal afferent projections follow a coarse viscerotopic organization in the NTS (82), yet individual vagal pathways and their NTS projections are not organized in a one-to-one manner (85). Intriguingly, a recent study integrating in vivo functional NTS imaging and viral tracing of vagal afferents showed that NTS responsiveness and vagal terminals are not perfectly correlated in anatomy (83), demonstrating additional complexity in signal processing. Not only the NTS subregions are organized in a viscerotopic manner [more anterior organs represented in the more rostrolateral area of the NTS (83)], but neurons in different NTS subregions also have different gene expression patterns. CRHR2^+^ neurons are more concentrated in the medial NTS and exhibit a higher rate of response to duodenum stretch, whereas TH^+^ neurons are more abundant in the lateral NTS, with a lower frequency of response to duodenum stretch (83). Furthermore, a subpopulation of epinephrine-positive NTS neurons were found to promote feeding, whereas another population expressing norepinephrine suppresses feeding (169), suggesting that information decoded in the NTS has different downstream targets and effects. Interestingly, in contrast to the spatial separation between signals from different organs, multiple sensory modalities from a single organ were observed to converge at the same subregion in the NTS (83), suggesting an early integration of interoceptive information across different modalities. The ability to discriminate among visceral spatial locations within the NTS is achieved through local inhibitory signals. When inhibition is blocked, NTS responses to certain stimuli in a single organ become broader (83). This might represent a more general principle in the interoceptive system, similar to touch sensation processing in the dorsal horn (170), that spatial discrimination is achieved through interneuron tuning in high-order brain regions.

The NTS conveys excitatory signals directly to autonomic output centers for rapid reflexes. The role of the NTS in the baroreflex is a good demonstration of its early convergence role for rapid autonomic control. When blood pressure elevation is sensed at the aortic arch and carotid sinuses and transmitted to the NTS via the glossopharyngeal and vagus nerves, the NTS directly recruits the nucleus ambiguus to slow down the heart rate (171). In parallel, the NTS projects to the caudal ventrolateral medulla to inhibit the sympathetic tone for vasodilation (172). Because the NTS integrates compositive information from different body regions, it is primed to work as a center for cross-organ regulation. NTS neurons innervate the adjacent dorsal motor nucleus of the vagus for vagal–vagal reflexes across organs along the gastrointestinal tract, such as the oesophagogastric or receptive relaxation reflex (173). Another example for cross-organ regulation is that the NTS receives information from hepatic vagal afferents and recruits the parasympathetic nerves to modulate the differentiation and maintenance of T regulatory cells in the gut (174). In addition to direct autonomic output, the NTS further sends processed information to third-order brain regions, including the paraventricular nucleus of the hypothalamus (175), parabrachial nucleus (PBN), and periaqueductal gray (PAG). These in turn are connected to higher brain centers, including the amygdala, insular cortex, rostral and anterior midcingulate, and orbitomedial prefrontal regions (176). Therefore, it is believed that the NTS’s function in perceiving and coordinating physiological responses is anatomically linked to higher-order brain functions such as perception, cognition, and adaptive behavior. However, how interoceptive information is processed within the NTS and distributed to higher-order brain regions remain to be elucidated.

Organs located in the lower regions of the body, such as the bladder, rectum, and reproductive organs, are only innervated by spinal sensory nerves and not by the vagus nerve (177). The spinal cord lamina I, which is responsible for transmitting temperature and pain sensations via the dorsal spinothalamic tract, has been suggested to function as a pathway for interoceptive information (178). Although the spinal circuitry for somatosensory processing has been extensively studied (179), how interoceptive information is integrated in the spinal cord remains largely unknown. Anatomically, nerve fibers from spinal cord lamina I project into various brainstem autonomic output nuclei, including the NTS, PBN, and PAG (178). The ventromedial posterior nucleus of the thalamus serves as a hub for information from the spinothalamic tract to the insular cortex. However, recent studies have revealed that stomach- (119) and rectum-innervating (78) DRG neurons project to specific spinal neurons located in lamina X. The organization of their central projection needs to be further characterized.

The PBN of the lateral hypothalamus receives interoceptive and exteroceptive inputs from the spinal cord and NTS (180). Anterograde tracing also showed that DRG neurons transmit gastric information to the PBN via the lateral reticular formation (119). The classical perspective on the PBN’s role is primarily focused on its involvement in aversion and avoidance behaviors. For example, PBN neurons can respond to intestinal GLP1 infusion and gut distension and mediate food reject as an output (119). However, there has been relatively little research exploring how the PBN processes positive valence (181). Gut-innervating neurons in the right nodose ganglion are linked to the substantia nigra through the NTS and PBN to induce dopamine release in the striatum and the subsequent rewarding effects, while the left vagus nerve may send more projections to the AP (182). PBN neurons are highly heterogeneous in both molecular and anatomical properties (183). Positive and negative valence of the interoceptive information might be coded by different PBN subregions: LiCl responses are limited to external-lateral PBN CGRP^+^ neurons that project to the amygdala, and CCK responsive neurons are mainly located in the dorsolateral and medial regions (182). This idea is also supported by anatomical evidence that the dorsolateral PBN sends projections through a ventral pathway to brain regions including the ventral tegmental area, whereas the external-lateral PBN projects to the amygdala and the insular cortex (183).

The insular cortex is the primary gustatory and visceral cortex and a multifunctional sensory center playing critical roles in a wide range of functions including emotion, decision making, self-awareness, social interactions, and language processing (81). It integrates both interoceptive and exteroceptive information. In humans, the right anterior insular cortex is found to represent the heartbeat awareness (184). Through a systemic whole-brain activity mapping in response to heart rate changes that was evoked using a novel optogenetic pacemaker tool in freely moving mice, the posterior insular cortex was identified as a potential region for cardiac interoceptive processing and tachycardia-induced anxiety (185). Similarly, in mice fear conditioning tests, heart rate drop during the freeze response reduces fear-induced activity in the insular cortex (186). It has been observed in humans that when nonpainful esophageal distention is presented along with pictures of fearful faces, there is an increase in insular cortex activation (187). These findings suggest that emotional and interoceptive states are integrated in the representation of subjective feelings at a given moment. A coarse viscerotopic map in the insular cortex, similar to the homunculus in the primary somatosensory cortex, has been elucidated. Interestingly, a clear anatomical segregation between visceral and gustatory signals does not exist in the insular cortex (81). Stimulation of the esophagus and stomach in humans initially activated the second somatosensory area and anterior cingulate cortex (ACC), whereas stimulation of the lower gut segments resulted in activation of the ACC and insular cortex (188). When performing nonpainful gastric distension, it was found that subjective feelings of fullness were linked to increased activity in the bilateral dorsal posterior insula, the left mid-insula, the left anterior insular cortex, and the ACC. In addition, cool temperatures might be encoded in a linear fashion in the dorsal posterior insula, but subjective ratings of these temperatures are linked to activity in the mid-insula and then more strongly correlated with activity in the anterior insular cortex (189). These results suggest a posterior-to-mid-to-anterior pattern of integration of interoceptive information (190).

## SPECIFIC FEATURES OF INTEROCEPTION AND BIOLOGICAL SOLUTIONS

Similar to exteroception, interoception is a complex process, including many subsystems sensing different signals. However, on the one hand, unlike the many well-characterized exteroceptive systems such as vision, audition, olfaction, and gustation that have a clear physiological purpose with well-defined signal modalities, an effective way to categorize interoceptive subsystems is still lacking. This is in part due to the special nature of interoceptive signals: In most cases the combination of physical modality and spatial location is required to determine its physiological role. For example, at the level of the vagus nerve, visceral organ, tissue layer, and signal modality are all coded in independent dimensions (85). On the other hand, compared to external signals, changes within the body are more predictable. Many characterized reflexes are simply achieved through hardwired circuits. Also, because signals are predictable, very efficient coding strategies can be used so that many fewer neurons are required to convey interoceptive information from the periphery to the CNS. Yet, these hardwired circuits contain multiple nodes (cell–cell connections) and, when necessary, they can be extensively regulated because each node can serve as a modulatory target. Also for the same reason, the computing demand for interoception is much less than for exteroception, and there is less need to generate conscious perceptions for interoceptive signals in most cases, consistent with the smaller dedicated brain volume and fewer cortical regions devoted to interoception.

It is also worth noting that sometimes it can be challenging to clearly define the boundary between interoception and exteroception. For example, sensing ingested food components via taste receptors expressed on the tongue belongs to one of the five basic external senses, whereas sensing digested nutrients and toxins or inhaled irritants via taste receptors expressed along the gastrointestinal tract or the airways is generally considered as interoception. Sometimes the distinction between interoception and somatosensation is also hard to define. Many cutaneous and muscular signals are generated from the body itself, such as inflammation-induced pain, chronic itch, and the movement of muscles that results in proprioception, and are thus more related to interoception than exteroception (1, 191–194).

Currently, the precise coding strategies for many primary interoceptive sensors still need to be determined. Applying state-of-the-art technologies such as single-cell spatial transcriptomics, high-resolution volumetric ultrastructural imaging, and real-time neural activity recordings in systematic cross-organ studies in the future will greatly advance our understanding of the coding logic of interoception.
